# ﻿*Polygalaspatulata* (sect. *Pseudosemeiocardium*, Polygalaceae), a new species from Guangxi, China

**DOI:** 10.3897/phytokeys.251.139955

**Published:** 2025-01-06

**Authors:** You Nong, Run-Hua Jiang, Qi-Min Hu, Xin-Cheng Qu, Xu-Chuan Gui, Gui-Yuan Wei, Li-Qun Lei

**Affiliations:** 1 Guangxi Key Laboratory of Traditional Chinese Medicine Quality Standards; Guangxi Institute of Chinese Medicine & Pharmaceutical Science, No. 20-1 Dongge Road, Nanning, Guangxi, China Guangxi Institute of Chinese Medicine & Pharmaceutical Science Nanning China; 2 Jilin Provincial Academy of Forestry Sciences, No. 3528 Linhe Street, Changchun, Jilin, China Jilin Provincial Academy of Forestry Sciences Changchun China; 3 Nanning Botanical Garden; Nanning Qingxiushan Scenic and Historic Tourism Development Co., Ltd, Nanning, Guangxi, China Nanning Botanical Garden Nanning China

**Keywords:** Karst cave, new species, *Polygala*, taxonomy

## Abstract

*Polygalaspatulata* Y. Nong & Run Hua Jiang (sect. Pseudosemeiocardium, Polygalaceae), a new species from a karst cave in west Guangxi, China, is described and illustrated. This new species resembles *Polygalaisocarpa* Chodat in its annual habit, terminal racemes, lamellate appendage, yellow flowers and glabrous seeds, but it can be easily distinguished by its sparsely white-pilose stem and leaf blades, spoon-shaped inner sepals, ovate outer sepals (ca. 1.5 mm) which is glandular and all persistent after anthesis, as well as its elliptic, reticulate seeds. Photographs, an illustration, a distribution map and a comparative table with the most similar species are also provided.

## ﻿Introduction

*Polygala* L. is one of the most diverse genera in the family Polygalaceae, with an almost global distribution (Anderson and Cronquist 1982). While the exact number of species remains contested due to ongoing discussion of genus delimitation, recent evaluations recognise between 422 and 552 species (Pastore et al. 2019; POWO 2024). Molecular phylogenetic studies consistently demonstrate *Polygala*’s polyphyletic nature, identifying three well-supported clades: a New World clade (NWC), an Old World clade (OWC) and a third clade comprising Polygalasubg.Chodatia (Eriksen 1993; Persson 2001; Forest et al. 2007; Pastore et al. 2019; Martinez et al. 2022). The OWC, the most species-rich clade, is distributed across Europe, Africa and Asia (Lyskov et al. 2019). Pastore et al. (2019) consider all NWC species as also part of Polygalasubg.Polygala. The *Polygala*NWC includes 213 species that may be divided into three sections: *Clinclinia* DC., *Monninopis* A.Gray, and *Timutua* DC. The *Polygala*OWC includes the type of the genus *Polygalavulgaris* L. and its ca.349 species are divided into 11 sections: *Blepharidium* DC., *Brachytropis* DC., *Chloropterae* (Chodat) Paiva, *Conosperma* Paiva, Leptaleae (Chodat) Paiva, *Madecassa* H.Perrier, *Megatropis* Paiva, *Microlophium* Spach, *Polygala*, *Psychanthus* (Raf.) DC. and *Tetrasepalae* (Chodat) Paiva.

Adema (1966) recognised 10 sections in *Polygala* and described a new section Pseudosemeiocardium Adema, later elevated to the status of subgenus by Chertk and Křísa (1977) and eventually segregated into the genus *Heterosamara* Kuntze emend. *Heterosamara* has been effectively used since Paiva (1998) and is recognisable by the heteropolar pollen with bilateral symmetry. Pastore et al. (2019) suggests for the first time that *Heterosamara* may not be monophyletic, forming a paraphyletic grade with Polygalasect.Chodatia and *Polygaloides* and before proposing formal taxonomic changes, their findings need to be further tested.

According to the Flora of China, species of Sect. Pseudosemeiocardium Adema are recognisable by annual herbs, often branching in corymbose fashion from the middle. Flowers are small, yellow, rarely purple-red, with one outer sepal persisting after flowering; seeds often have papillary or helmet-shaped caruncles. About seven species are distributed across the southern slopes of the Himalayas, northern India, the Indochina Peninsula, Malaysia, the Philippines, Indonesia, western Papua New Guinea and Japan. Five species are native to China, distributed throughout the country, but most abundant in the southwest (Chen et al. 1997).

Forty-two species and eight varieties of *Polygala* were found in China, widely spread across the country, but most abundant in the south-western and southern regions (Chen et al. 1997), 44 species of *Polygala* were recognised, with 21 endemic to China in the flora of China (Chen et al. 2008), but most recently, one new species was identified from China (Liu et al. 2024). During our field surveys in Bama County, Guangxi in August 2024, we discovered a special *Polygala* population in a karst cave, this population of plants resembling *Polygalaisocarpa* Chodat in being an annual herb with terminal racemes, lamellate appendage, yellow flowers and glabrous seeds. However, it differs distinctively by its sparsely white-pilose stem and leaf blade, with outer sepals remaining persistent after anthesis. After consulting relevant literature (Chen et al. 1997; Chen et al. 2008; Liu et al. 2024) and checking relevant specimens, we confirm that this unusual plant represents a new *Polygala* species, which we describe and illustrate below.

## ﻿Materials and methods

The new species was described, based on field observations that were made in August 2024 and examination of herbarium specimens at GXMI. Other related *Polygala* species were examined, based on online images from the Kew Herbarium Catalogue (http://apps.kew.org/herbcat/gotoHomePage.do) and JSTOR Global Plants (http://plants.jstor.org/). Morphological characters that distinguish it from all other species in the genus of *Polygala* are used. We also observed living plants of the new species at flowering and fruiting time (August and September). We observed characters of stem, petiole, leaf, racemes, pedicel, bracts, sepals, petals, stamens, ovary, style, capsule and seeds.

Descriptions were written, based on herbarium specimens. Measurements were made with a tape measure and callipers. The structure of the indumentum and its distribution were observed and described under a dissecting microscope at magnifications of more than 20×. Additional information on locality, habitat, ecology, plant form and fruits were collected in the field and taken from herbarium labels. The conservation threat assessment followed IUCN Categories and Criteria (IUCN 2022).

## ﻿Results and discussion

### ﻿Taxonomy

#### ﻿ Polygala spatulata

Taxon classificationPlantaeFabalesPolygalaceae

Y.Nong & Run Hua Jiang sp. nov.

E1406839-E7F4-5A3E-922F-02E760BD7A5B

urn:lsid:ipni.org:names:77354775-1

**Figure 1.**
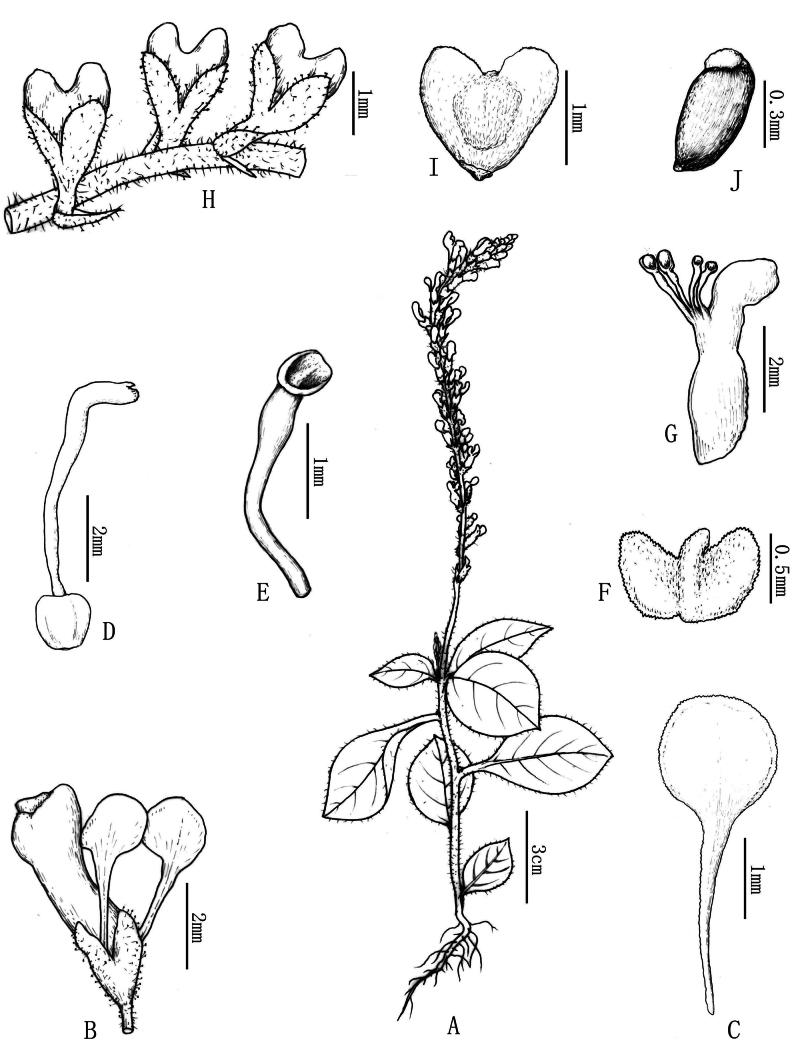
Line drawing of *Polygalaspatulata Y. Nong & Run Hua Jiang***A** flowering plant **B** flower **C** inner sepal **D** style **E** stamen **F** appendage **G** longitudinal section of corolla **H** fruiting branch **I** capsule **J** seed (Drawn by Xin-cheng Qu).

**Figure 2.**
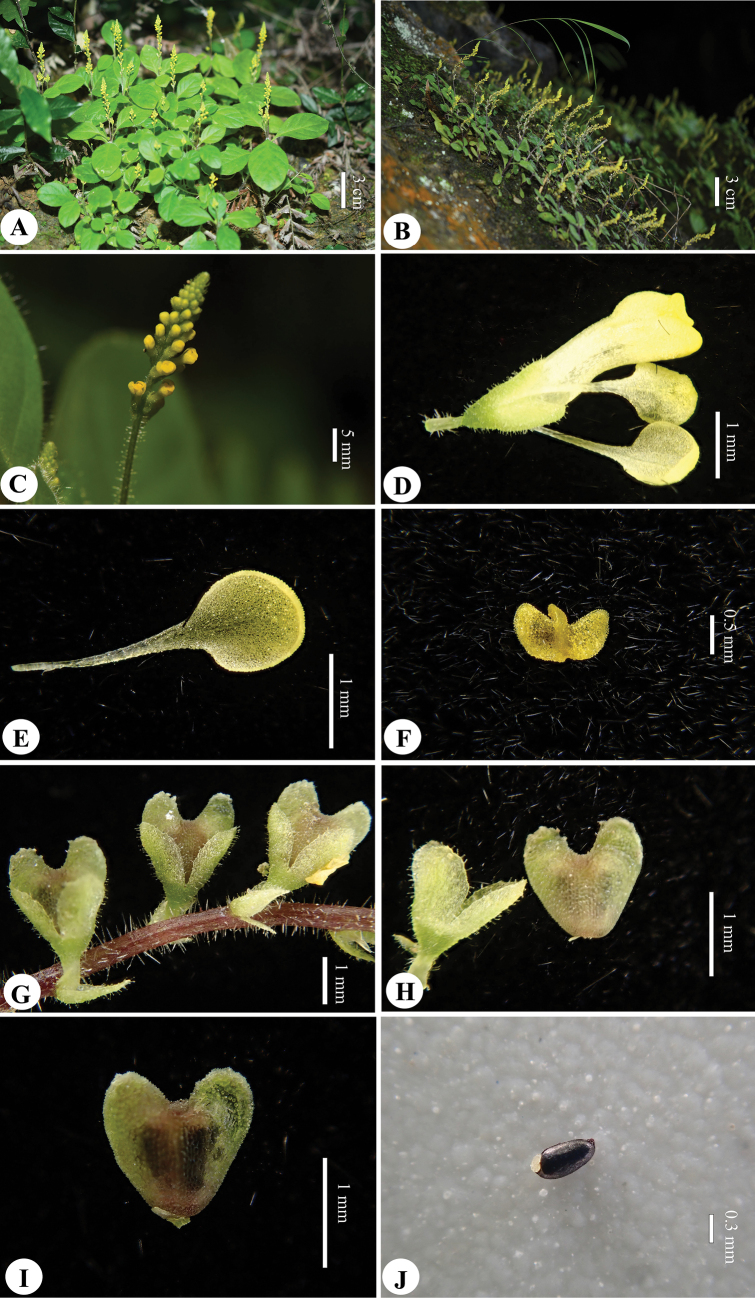
*Polygalaspatulata Y. Nong & Run Hua Jiang***A**, **B** habitat **C** racemes **D** flower **E** inner sepal **F** appendage **G** fruiting branch **H** capsule and outer sepal **I** capsule **J** seed (Photographed and edited by You Nong).

**Figure 3.**
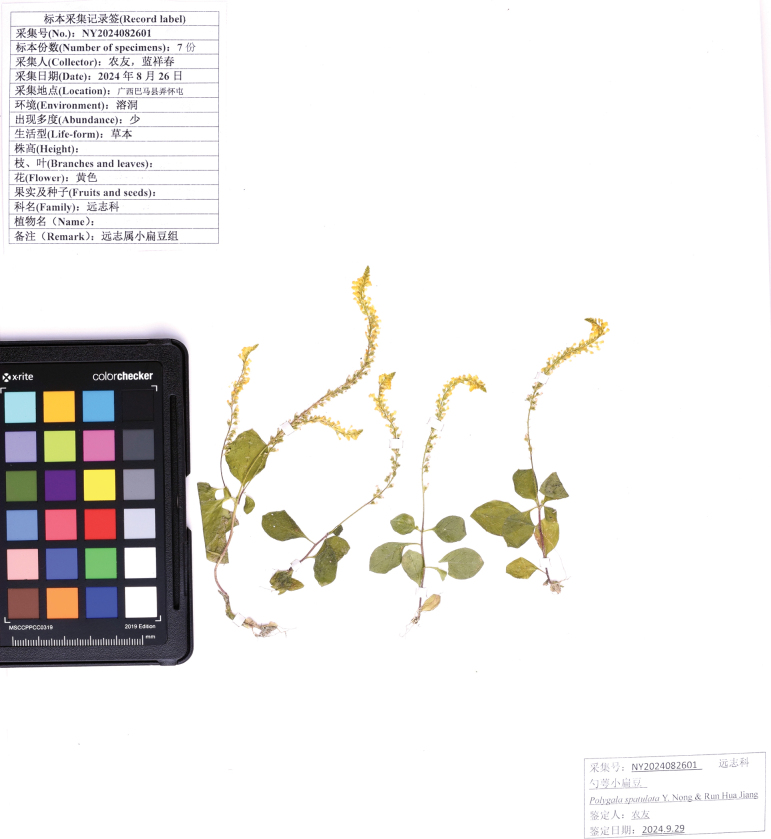
The type specimen of *Polygalaspatulata Y. Nong & Run Hua Jiang*.

**Figure 4.**
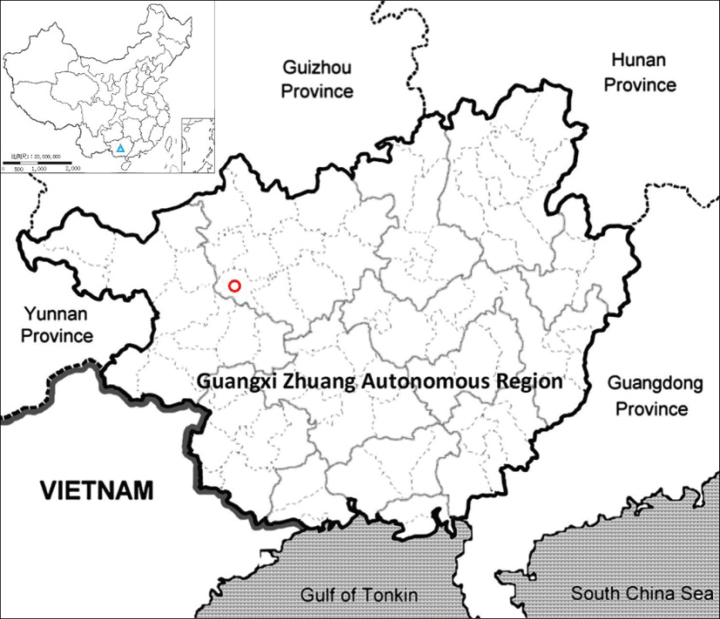
The distribution of *Polygalaspatulata Y. Nong & Run Hua Jiang* (red circle) in Guangxi, China.

Figs 1 , 2 , 3 , 4 Chinese name: “sháo è xiǎo biǎn dòu”(勺萼小扁豆)

##### Diagnosis.

*Polygalaspatulata* is most similar to *P.isocarpa* Chodat, but it can be easily distinguished by its stems sparsely white pilose (vs. glabrous), leaf blade oval or obovate, membranous, abaxially and adaxially sparsely white pilose, apex round or obtuse (vs. ovate or ovate-triangular, papery, both surfaces glabrous or only abaxially sparsely setose, apex acuminate), its inner sepals spoon-shaped (vs. oblong or ovate) and its outer sepals 3, ovate, ca. 1.5 mm, glandular, all persistent after anthesis (vs. outer sepals 3, broadly ovate, ca. 1 mm, glabrous, one persistent after anthesis).

##### Type.

China • Guangxi: Bama County, 23°08'46"N, 107°03'22"E, alt. 530 m, in a cave, 26 August 2024, *Y Nong NY2024082601* (GXMI). (***Holotype***: 051192 GXMI!; ***isotypes***: IBK!).

##### Description.

Herbs annual, erect, 5–15 cm tall. Stems terete, sparsely white pilose, 1–3 branches, apically; branchlets spreading, slender. Petiole 0.5–1.5 cm, sparsely white pilose; leaf blade oval or obovate, 3–7 × 2–3 cm, membranous, abaxially and adaxially sparsely white pilose, mid-vein slightly raised abaxially and adaxially, lateral veins 4–5 pairs, anastomosing near margin, base cuneate, decurrent, apex round or obtuse. Racemes terminal, sparsely white pilose, 3–10 cm. Pedicel ca. 1 mm, slender; bracts 3, caducous, subulate, unequal, glandular. Sepals 5; outer sepals 3, ovate, ca. 1.5 mm, glandular outside, all persistent after anthesis; inner sepals 2, spoon-shaped, 1–1.5 mm, glabrous, base unguiculate, apex rounded. Petals 3, connate in lower 1/2, yellow; lateral petals rectangular-oblong, longer than keel; keel with apex broadly retuse, with appendage of 2 lobes. Stamens 8; filaments lower 1/3 united, forming an open staminal sheath; anthers ovoid. Ovary subglobose, 0.8–1.0 mm in diam.; style gradually broadening from base towards apex, curved, apex funnel-form; stigma at lower margin. Capsule obcordate, glabrous, 1–1.2 mm in diam., narrowly winged. Seeds black, shiny, elliptic, 0.6 × 0.3 mm, reticulate, glabrous; strophiole white, 2-lobed.

##### Etymology.

The specific epithet “*spatulata*” refers to the inner sepals 2, spoon-shape of the new species.

##### Distribution and habit.

Currently, *Polygalaspatulata* is known only from the northwest of Guangxi, China (Fig. 4). It has been mainly found in a karst cave at an elevation of about 530 m.

##### IUCN red list category.

Due to limited available data, the conservation status of this new species cannot be definitively assessed. Following IUCN Criteria (IUCN 2022), it is currently classified as Data Deficient (DD) pending further research and information.

### ﻿Comparision with other *Polygala* species

*P.spatulata* is similar to *P.umbonata* Craib, but it can be easily distinguished by its stems sparsely white pilose (vs. glabrous), its leaf blade oval or obovate, membranous, abaxially and adaxially sparsely white pilose, apex round or obtuse (vs. ovate or elliptic to elliptic-lanceolate, papery, abaxially glabrous, adaxially sparsely white setose or along margin densely minutely setose, apex acuminate), its ovary subglobose, 0.8–1.0 mm in diam. (vs. ellipsoidal, ca. 0.5 mm in diam.) and its seeds reticulate, glabrous (vs. tuberculate, sparsely white pilose). More detailed morphological differences amongst the three similar species are shown in Table 1.

**Table 1.** Main morphological differences amongst *Polygalaspatulata*, *P.isocarpa* and *P.umbonata*.

Morphological traits	*Polygalaspatulata*	*P.isocarpa*	*P.umbonata*
**Height**	5–15 cm	5–14 cm	15–20 cm
**Stems**	sparsely white pilose	glabrous	glabrous
**Petiole**	0.5–1.5 cm, sparsely white pilose	ca. 5 mm, winged, glabrous	ca. 1 cm, glabrous
**Leaf blade**	oval or obovate, 3–7 × 2–3 cm, membranous, abaxially and adaxially sparsely white pilose, apex round or obtuse	ovate or ovate-triangular, 1.5–2.5 × 0.6–1 cm, papery, both surfaces glabrous or only abaxially sparsely setose, apex acuminate	ovate or elliptic to elliptic-lanceolate, 3–4 × 1.5–2 cm, papery, abaxially glabrous, adaxially sparsely white setose or along margin densely minutely setose, apex acuminate
**Racemes**	terminal, 3–10 cm	terminal, ca. 7 cm	terminal or axillary, 1–4 cm
**Bracts and bracteoles**	subulate, glandular	ovate, glabrous	subulate, glabrous
**Sepals**	outer sepals 3, all persistent after anthesis, ovate, ca. 1.5 mm, glandular; inner sepals 2, petaloid, orbiculate or obovate, 1–1.5 mm, base unguiculate, apex rounded	outer sepals 3, one persistent after anthesis, broadly ovate, ca. 1 mm; inner sepals 2, oblong or ovate, ca. 2 × 1.5 mm, 3-veined, base unguiculate, apex rounded	outer sepals 3, caduceus, ovate, ca. 1.5 mm; inner sepals 2, petaloid, elliptic, 3–3.5 mm, base unguiculate, apex rounded
**Ovary**	subglobose, 0.8–1.0 mm in diam.	obovoid or subglobose, ca. 1 mm in diam.	ellipsoidal, ca. 0.5 mm in diam.
**Capsule**	obcordate, glabrous, 1–1.2 mm in diam., narrowly winged	broadly obcordate or suborbicular, ca. 2 mm in diam., narrowly winged	suborbicular, ca. 3 mm in diam., winged
**Seeds**	black, shiny, elliptic, 0.6 × 0.3 mm, reticulate, glabrous	black, shiny, ovoid, ca. 1 mm in diam., tuberculate, glabrous	black, elliptic, ca. 1 mm in diam., tuberculate, sparsely white pilose

## Supplementary Material

XML Treatment for Polygala spatulata

## ﻿Additional information

### Author ORCIDs

You Nong https://orcid.org/0000-0001-7004-0946

Run-Hua Jiang https://orcid.org/0009-0005-8619-2836

Qi-Min Hu https://orcid.org/0009-0003-0490-3557

Xin-Cheng Qu https://orcid.org/0009-0009-9078-9976

Xu-Chuan Gui https://orcid.org/0009-0000-6263-3821

Gui-Yuan Wei https://orcid.org/0000-0003-0652-1213

Li-Qun Lei https://orcid.org/0009-0009-7319-5439

### Data availability

All of the data that support the findings of this study are available in the main textn.
